# Oncogenic osteomalacia associated with mesenchymal tumor in the middle cranial fossa: a case report

**DOI:** 10.1186/1752-1947-6-181

**Published:** 2012-07-02

**Authors:** Isao Chokyu, Kenichi Ishibashi, Takeo Goto, Kenji Ohata

**Affiliations:** 1 Department of Neurosurgery, Osaka City University Graduate School of Medicine, 1-4-3 Asahi-machi, Abeno-ku, Osaka, 545-8585, Japan

## Abstract

**Introduction:**

Tumor-induced osteomalacia is a paraneoplastic syndrome of hypophosphatemia. Osteomalacia causes multiple bone fractures and severe pain.

**Case presentation:**

We report the case of a 57-year-old Japanese man with tumor-induced osteomalacia associated with a middle cranial fossa bone tumor. The tumor was successfully resected by using a middle fossa epidural approach. His phosphate level recovered to a normal range immediately after the surgery.

**Conclusions:**

It is rare that tumor-induced osteomalacia originates from the middle skull base. This report suggests that, if patients have a clinical and biochemical picture suggestive of tumor-induced osteomalacia, it is crucial to perform a meticulous examination to detect the tumor or the lesion responsible for the tumor. The serum level of fibroblast growth factor 23 is the most reliable marker for evaluating the treatment outcome of tumor-induced osteomalacia.

## Introduction

Tumor-induced osteomalacia is a rare acquired disorder. It has been reported to occur in patients with hypophosphatemia because of excessive renal phosphate excretion secondary to various types of mesenchymal tumors, including hemangiopericytoma, giant cell tumor, and fibroma. Patients typically present with a history of chronic bone pain, fractures, and proximal motor weakness. Children may exhibit poor growth and lower-extremity deformity. The tumors are often benign, small, and difficult to detect. A recent report has suggested that fibroblast growth factor 23 (FGF-23) is the most reliable marker for the detection of these tumors [1].

We report a case of tumor-induced osteomalacia associated with a middle fossa bone tumor. The tumor was successfully resected via a middle fossa epidural approach. Phosphate level recovered to a normal range immediately after the surgery. The diagnostic evaluation, etiology of hypophosphatemia, and treatment are discussed.

## Case presentation

**Figure 1:**
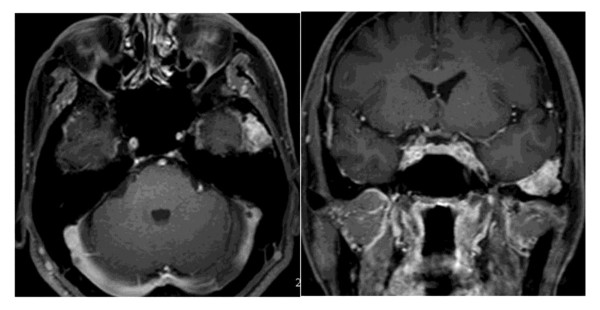
Axial and coronal magnetic resonance images with contrast show a left epidural mass at the middle cranial fossa.

**Figure 2:**
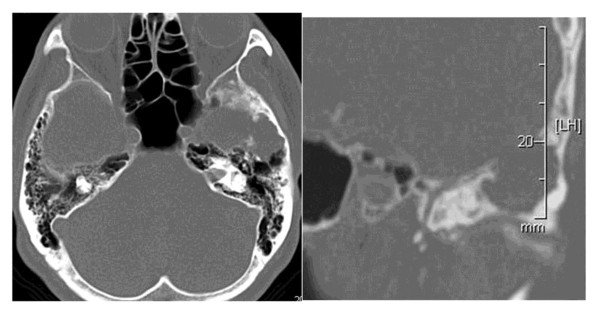
Bone computed tomography images demonstrate a bone-eroded lesion at the middle cranial fossa.

A 57-year-old Japanese man was initially referred to our hospital for treatment of multiple bone fractures. In 2007, he had fallen down and fractured his foot. Fortunately, he had recovered with conservative therapy; however, the delay of bone fusion in his case was notable. In 2008, he experienced a sudden onset of costal pain with trivial trauma, and multiple costal fractures were diagnosed. The costal pain did not resolve during these two years. Two years after his initial foot fracture, our patient exhibited a serum phosphorus concentration of 1.9mL/dL (normal range is 2.5 to 4.5mg/dL) and a serum calcium concentration of 9.0mg/dL (normal range is 8.5 to 10.5mg/dL). Furthermore, a detailed investigation revealed persistent hypophosphatemia and a serum 1,25-dihydroxyvitamin D concentration of 18pg/mL (normal range is 25 to 50pg/mL). Serum levels of FGF-23 were elevated to 84pg/mL (normal range is 10 to 50pg/mL). Subsequent magnetic resonance (MR) images revealed a tumor in the middle cranial base; the tumor reached the temporomandibular joint and was 27 × 18 × 20mm in size. This tumor was clearly and homogeneously enhanced on gadolinium MR images (Figure 1). Bone computed tomography scans showed destructive change to bone up to the external cortical layer in the middle cranial base (Figure 2). We presumed that hypophosphatemia and vitamin D deficiency were related to this tumor.

**Figure 3:**
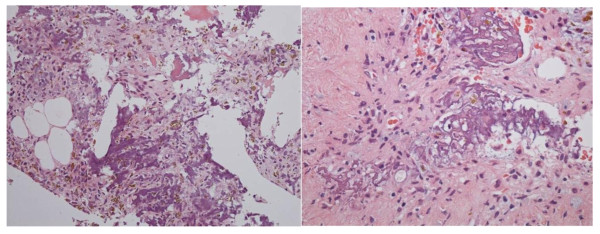
A photomicrograph of the resected tumor shows small rounded spindled cells with a smudgy matrix (hematoxylin and eosin, or H-E).

Two months after the MR images were taken, we performed the tumor resection via a middle fossa epidural approach. The tumor was located and eroded in the middle fossa cranial base and was easy to remove by curretting. The tumor had adhered to the dura mater in some parts but did not invade the subdural space. Finally, the tumor was totally resected, preserving the temporomandibular joint. Histopathological diagnosis was consistent with a phosphaturic mesenchymal tumor that showed round or spindled cells embedded in a smudgy blue-gray material (Figure 3).

**Figure 4:**
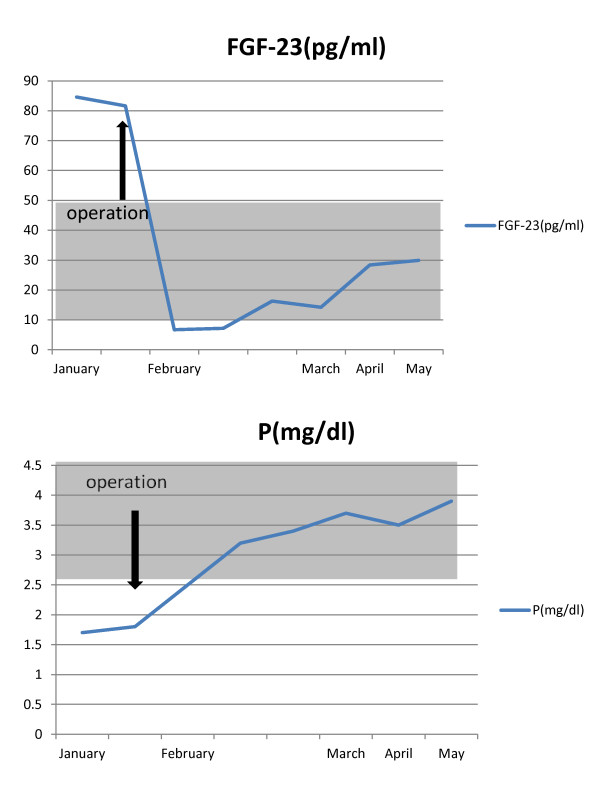
**Charts display pre-and post-operative laboratory dates.** OP, operation. P, phosphorous level. FGF-23, fibroblast growth factor 23 level.

**Figure 5:**
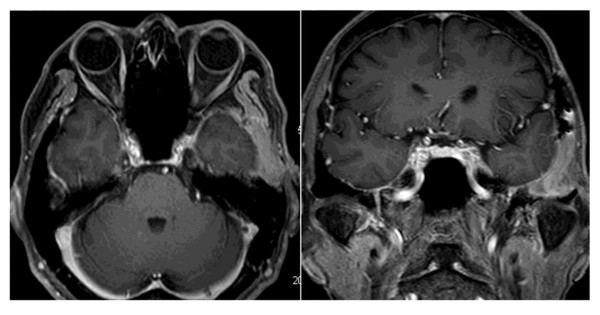
Magnetic resonance images with contrast one year after surgery reveal no enhanced region.

The post-operative course was uneventful. The hypophosphatemia and vitamin D deficiency resolved five days after the surgery. Serum levels of FGF-23 decreased to normal (14pg/mL) seven days after the surgery (Figure 4). Costal pain in our patient was completely resolved one month after the surgery. MR images at one-year follow-up showed no recurrence of the tumor, and his serum phosphate level was normal (Figure 5).

## Discussion

Tumor-induced osteomalacia is an uncommon condition. These tumors are usually mesenchymal or mixed connective tissue arising from either soft tissues or bone [2]. They are benign in nature. Even in histologically malignant tumors, local recurrence or distant metastasis is extremely rare. The most common types of these tumors are hemangiopericytomas. Other types include fibromas, chondrosarcomas, neuroblastomas, and prostate carcinomas [3,4]. The first case was reported by McCance [5] in 1947. In 1995, Crouzet and colleagues [6] reviewed 100 cases reported in the medical literature. Since then, there have been many other case reports about oncogenic osteomalacia. Gonzalez-Compta and colleagues [7] reviewed 21 cases of osteomalacia induced by head and neck tumors. The authors reported that 57% of these tumors were located in the sinonasal area and that the mean age at diagnosis was 45 years. There has been no finding of gender predominance in the previous reports. Bone pain, muscle weakness, fractures, skeletal deformities, and gait disturbance are the most common clinical symptoms. This slow-growing tumor can occur in unusual sites in the body. Pirola and colleagues [8] reported oncogenic osteomalacia of the thoracic spine. However, oncogenic osteomalacia originating from the middle cranial fossa is very rare.

Biochemical analysis often detects abnormalities in serum chemical concentrations. Generally, those patients who have hypophosphatemia have normal calcium levels and a low 1,25-dihydroxyvitamin D concentration. These imbalances resolve after total surgical exision of the tumor. The serum phosphate level is basically regulated by intestinal phosphate absorption, renal phosphate excretion, and dynamic equilibrium between circulatory phosphate and intracellular phosphate or phosphate in calcified bone. Of these, renal phosphate excretion is believed to be the main regulator of the chronic phosphate level. Recently, the mechanism of tumor-induced osteomalacia was thought to be secondary to inhibition of the ability of the renal tubule to reabsorb phosphorus and activate calcitriol synthesis. These processes lead to hypophosphatemia. The most reliable marker for the detection of tumor-induced osteomalacia is FGF-23 [9], which is a secreted peptide hormone overexpressed by the tumor in patients with tumor-induced osteomalacia. Fukumoto [10] reported that FGF-23 suppresses phosphate reabsorption by decreasing expression levels of the type 2a and 2c sodium phosphate co-transporter in the brush border membrane of proximal tubules. At the same time, FGF-23 reduces serum 1,25-dihydroxyvitamin D levels in part by suppressing 1,25-dihydroxyvitamin D production. As 1,25-dihydroxyvitamin D enhances intestinal phosphate absorption, FGF-23 decreases the serum phosphate level partly by reducing intestinal phosphate absorption through suppressing the serum 1,25-dihydroxyvitamin D level. Symptoms resolve once the tumor is totally removed. Therefore, it is necessary to consider the total excision of the tumor as a treatment strategy.

## Conclusions

We treated a case of hypophosphatemia associated with tumor-induced osteomalacia. The tumor was successfully resected by using a middle fossa epidural approach. Phosphate levels recovered to normal immediately after the surgery. For patients who have a clinical and biochemical profile suggestive of tumor-induced osteomalacia, it is crucial to perform an in-depth examination to detect the tumor or the lesion responsible for the tumor. FGF-23 is the most reliable marker to evaluate the usefulness of any treatment for tumor-induced osteomalacia.

## Consent

Written informed consent was obtained from the patient for publication of this case report and any accompanying images. A copy of the written consent is available for review by the Editor-in-Chief of this journal.

## Abbreviations

FGF-23: Fibroblast growth factor 23; MR: Magnetic resonance.

## Competing interests

The authors declare that they have no competing interests.

## Authors’ contributions

IC was involved in the diagnosis and treatment of our patient and wrote the manuscript. KI, TG, and KO were involved in the diagnosis of our patient and helped to revise the manuscript. All authors read and approved the final manuscript.

## References

[B1] de Beur Jan SM Tumor-induced osteomalacia JAMA 2005 294 1260 1267 10.1001/jama.294.10.1260 16160135

[B2] David K Revesz T Kratimenos G Krausz T Crockard HA Oncogenic osteomalacia associated with a meningeal phosphaturic mesenchymal tumor. Case report J Neurosurg 1996 84 288 292 10.3171/jns.1996.84.2.0288 8592237

[B3] Folpe AL Fanburg-Smith JC Billings SD Bisceglia M Bertoni F Cho JY Econs MJ Inwards CY de Beur Jan SM Mentzel T Montgomery E Michal M Miettinen M Mills SE Reith JD O’Connell JX Rosenberg AE Rubin BP Sweet DE Vinh TN Wold LE Wehrli BM White KE Zaino RJ Weiss SW Most osteomalacia-associated mesenchymal tumors are a single histopathologic entity: an analysis of 32 cases and a comprehensive review of the literature Am J Surg Pathol 2004 28 1 30 10.1097/00000478-200401000-00001 14707860

[B4] Kumar R Tumor-induced osteomalacia and the regulation of phosphate homeostasis Bone 2000 27 333 338 10.1016/S8756-3282(00)00334-3 10962341

[B5] McCance RA Osteomalacia with Looser’s nodes (Milkman’s syndrome) due to a raised resistance to vitamin D acquired about the age of 15 years Q J Med 1947 16 33 46 20296654

[B6] Crouzet J Mimoune H Beraneck L Juan LH Hypophosphatemic osteomalacia with plantar neurilemoma. A review of the literature (100 cases) Rev Rhum Engl Ed 1995 62 463 466 7552213

[B7] Gonzalez-Compta X Manos-Pujol M Foglia-Fernandez M Peral E Condom E Claveguera T Dicenta-Sousa M Oncogenic osteomalacia: case report and review of head and neck associated tumours J Laryngol Otol 1998 112 389 392 9659507 10.1017/s0022215100140551

[B8] Pirola E Vergani F Casiraghi P Leone EB Guerra P Sganzerla EP Oncogenic osteomalacia caused by a phosphaturic mesenchymal tumor of the thoracic spine J Neurosurg Spine 2009 10 329 333 10.3171/2009.1.SPINE08351 19441990

[B9] Carpenter TO Oncogenic osteomalacia–a complex dance of factors N Engl J Med 2003 348 1705 1708 10.1056/NEJMe030037 12711747

[B10] Fukumoto S Fibroblast growth factor (FGF) 23 works as a phosphate-regulating hormone and is involved in the pathogenesis of several disorders of phosphate metabolism [in Japanese] Rinsho Byori 2007 55 555 559 17657990

